# Delayed Onset Type I Allergic Reaction Following Black Tattoo Removal Using Picosecond Laser

**DOI:** 10.1097/DSS.0000000000004127

**Published:** 2024-03-13

**Authors:** Suzanne van Santen, Esther J. van Zuuren, Albert Wolkerstorfer, Sebastiaan A.S. van der Bent

**Affiliations:** * Department of Dermatology, Leiden University Medical Center, Leiden, the Netherlands; † Department of Dermatology, Amsterdam University Medical Center, Amsterdam, the Netherlands; ‡ Tattoo Clinic (Tattoo poli), Department of Dermatology, Alrijne Hospital, Leiden, the Netherlands

Picosecond lasers are increasingly used as the mainstay laser technology alongside nanosecond lasers for removing tattoos. We present a unique case of a patient who developed progressive and recurrent Type I allergic reactions 1 week following each laser treatment.

A 20-year-old female patient with no prior medical conditions was cosmetically dissatisfied with a dark section of her arm sleeve tattoo and requested laser treatment to lighten it. She underwent her first laser treatment with a picosecond laser (PicoWay, Syneron Candela, 1,064 nm, 0.8 Joules/cm^2^, 4 Hertz, spot size 6 mm) and developed blisters in the days following the procedure, which resolved spontaneously. Eight days after the treatment, she experienced severe itching, burning, redness, and swelling in the treated area with sharply defined keratotic plaques, which spontaneously recovered (Figure 1). Similarly, the second laser treatment (same device, 0.6 Joules/cm^2^) 2 months later resulted in localized blistering during the first days, but after 8 days, she experienced facial angioedema and widespread urticaria on the body. When seeking medical assistance, she collapsed but regained consciousness spontaneously at her physician's clinic. Treatment with prednisone and antihistamines led to a gradual recovery of her angioedema and urticaria. The third laser treatment (same device, 0.9 Joules/cm^2^) 3 months later followed a similar pattern with initial local blistering. On day 7, she developed unbearable itching on her arm and regularly used ice packs for cooling. The next day she developed severe facial angioedema, causing considerable difficulty in opening her eyes, angioedema on the hands, wrists, and feet, and laryngeal edema resulting in difficulty swallowing. Again, widespread urticaria reappeared (Figure 2), without cardiovascular symptoms or syncope. Treatment with prednisone and antihistamines did not provide immediate relief, leaving the patient with severe urticaria for 2 days; after 10 days, she was fully recovered. No subsequent allergy testing was conducted because no reliable skin tests are available regarding tattoo pigments or their breakdown products.

**Figure 1.**
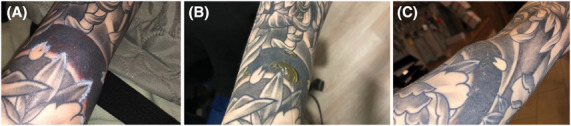
Cutaneous reactions after first laser treatment (A), blistering first days after first treatment (B), and local swelling 1 week after treatment (C).

**Figure 2.**
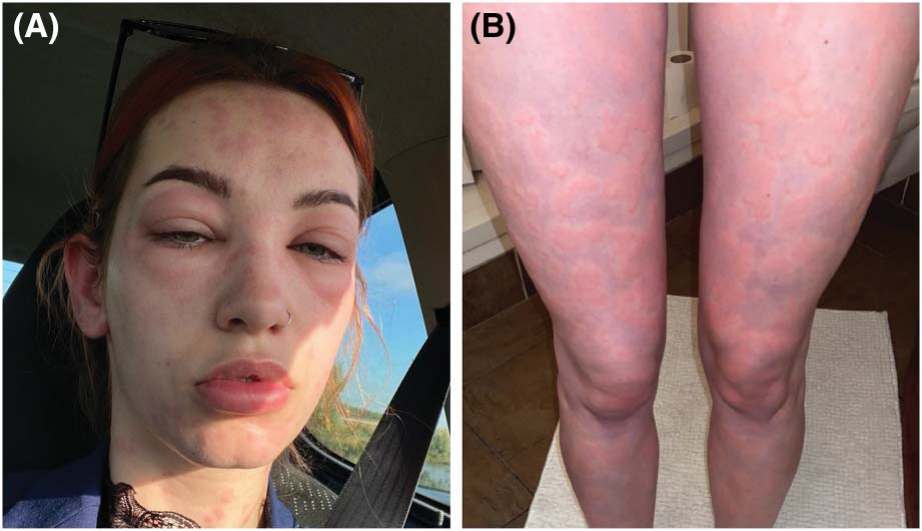
Facial angioedema (A) and urticaria on the legs (B) after third laser treatment.

## Discussion

Laser treatment is widely regarded as the most effective method for tattoo removal with up to 5.8% of all tattoo complications in a hospital setting being related to tattoo removal.^1^ Alongside the use of nanosecond lasers, the use of picosecond lasers is rising as a mainstay device for tattoo removal. However, the literature reporting adverse reactions associated with their use is scarce. It is known that laser-assisted tattoo removal has the potential to produce a range of degradation products, which can be linked to human toxicity or hypersensitivity reactions, typically manifesting as delayed Type IV allergic cutaneous reactions.^2^ Although rare, immediate Type I allergic reactions have been reported in the context of nanosecond laser usage and included itch, swelling, redness, and localized urticaria occurring within minutes to hours after the laser sessions, conform the time span of a Type I reaction.^3^ It has been suggested that these reactions are due to the extracellular release of tattoo pigment fragments, directly triggering an immune response.

However, in our case, we suspect that laser treatment caused a delayed onset Type I allergic reaction to one of the breakdown products or metabolites, which was released into the blood circulation approximately 1 week after the treatment.

Symptoms indicative for a Type I allergic reaction included the limited local complaints upon first laser treatment and the progressive course of widespread urticaria, angioedema, and laryngeal edema after the 2 successive laser treatments. A delayed onset of these symptoms with an exact equal time span of 1 week was repeatedly observed after each laser treatment.

During laser tattoo removal, tattoo pigments break down into smaller particles as a result of photomechanical interaction and can be engulfed and eliminated through phagocytosis, lymphatic drainage, and may undergo hepatic metabolization.^4^ Moreover, tattoo pigments can also be metabolized directly in the skin by cytochrome P450 and transferred toward regional lymph nodes and the circulatory system.^4^ We hypothesize that one of these routes are involved in the delayed onset of allergic symptoms in our case. However, whether the timespan corresponds with average rates of lymphatic clearance of breakdown products or formation of metabolites is currently unknown. One similar case report describes an urticarial and systemic anaphylactic reaction 3 days after laser treatment with a 755-nm picosecond alexandrite laser of a multicolored tattoo.^5^ Current literature on the metabolism of (degraded) tattoo pigments remains scarce and has mainly focused on azo-based tattoo pigments associated with yellow-, orange-, and red-colored tattoos, rather than black inks.^4^ Pretreatment with antihistamines and topical and oral corticosteroids have been reported to prevent local Type I allergic reactions; the efficacy of these interventions in patients experiencing delayed progressive systemic allergic symptoms is unknown. We advised our patient to discontinue further laser treatments to prevent potential worsening of the allergy. Up to now, 2 delayed onset Type I allergic reactions have been reported after picosecond laser-assisted tattoo removal. However, it is currently unknown whether these types of reactions may similarly occur on breakdown products of nanosecond lasers.

## Conclusion

We reported a rare case of a delayed onset Type I allergic reaction following black tattoo removal using picosecond laser. Existing literature on allergic reactions resulting from laser-assisted tattoo removal is limited, especially for picosecond lasers, highlighting the need for further research to enhance our understanding of the prevalence, risk factors, causative allergens, and optimal management for these reactions. Although rare, delayed onset of a Type I allergic reaction may occur after laser treatment and clinicians should be aware of these reactions when counselling patients undergoing laser-assisted tattoo removal.
